# Nutrition, Hygiene and Stimulation Education for Impoverished Mothers in Rural Uganda: Effect on Maternal Depression Symptoms and Their Associations to Child Development Outcomes

**DOI:** 10.3390/nu11071561

**Published:** 2019-07-11

**Authors:** Prudence Atukunda, Grace K. M. Muhoozi, Ane C. Westerberg, Per O. Iversen

**Affiliations:** 1 Department of Nutrition, Institute of Basic Medical Sciences, University of Oslo, 0372 Oslo, Norway; 2 Department of Human Nutrition and Home Economics, Kyambogo University, P.O. Box 1, 256 Kyambogo, Uganda; 3 Institute of Health Sciences, Kristiania University College, Kirkegata 24 Oslo, Norway; 4 Department of Hematology, Oslo University Hospital, 0372 Oslo, Norway; 5 Division of Human Nutrition, Faculty of Medicine and Health Sciences, Stellenbosch University, 7505 Tygerberg, South Africa

**Keywords:** children, complementary feeding, developmental outcomes, group dynamics theory, maternal depression, nutrition education

## Abstract

Optimal nutrition improves child development, and impaired development is associated with maternal depression symptoms, in particular in low resource settings. In this follow-up of an open cluster-randomized education trial, we examined its effects among mothers in rural Uganda on their depression symptoms and the association of these symptoms to child development. The education comprised complementary feeding, stimulation, and hygiene. We assessed 77 intervention mothers and 78 controls using Beck Depression Inventory-II (BDI-II) and Center for Epidemiologic Studies Depression Scale (CES-D) scores. Child development was assessed with Bayley Scales of Infant and Toddler Development-III (BSID-III) composite scores for cognitive, language and motor development. Compared to controls, the intervention reduced depression symptoms’ scores with mean (95% CI) differences: −8.26 (−11.49 to −1.13, *p* = 0.0001) and −6.54; (−8.69 to −2.99, *p* = 0.004) for BDI II at 20–24 and 36 months, respectively. Similar results were obtained with CES-D. There was a negative association of BDI-II scores and BSID-III cognitive and language scores at 20–24 (*p* = 0.01 and 0.008, respectively) and 36 months (*p* = 0.017 and 0.001, respectively). CES-D associations with BSID-III cognitive and language scores showed similar trends. BSID-III motor scores were associated with depression scores at 36 months for both BDI-II and CES-D (*p* = 0.043 and 0.028, respectively). In conclusion, the group education was associated with reduced maternal depression scores. Moreover, the depression scores were inversely associated with child cognitive and language development outcomes.

## 1. Introduction

Early childhood is characterized by rapid cognitive and social development changes that require optimal nutrition [1]. Introduction of complementary feeding involves a gradual transition from breastfeeding to eating foods and liquids along with breast milk when breast milk alone is no longer sufficient to meet the nutritional requirements of infants [2]. This promotes adequate nutritional and developmental achievements in infancy [3].

Inadequate infant and young child feeding practices increase the risk of morbidity and mortality, especially in low resource settings. Moreover, childhood morbidity has been associated with maternal depression symptoms in such settings [4]. The prevalence of postnatal maternal depression symptoms in 2015 was 50% in South Africa [5] while in Zimbabwe the prevalence increased from 16 to 34% between 1995 and 2015 [6]. A systematic review from 2019 indicated that maternal depression symptoms contributed to 20% of postpartum deaths [7]. Furthermore, maternal depression symptoms are also common among women with small children [8]. Depression during the time of introduction of complementary foods compromises the mothers’ ability to make adequate and nutritious food for the child [9]. In line with this, mothers’ depressive symptoms markedly affect their ability to attend to children’s feeding practices and cognitive development, henceforth placing their children at risk of delayed developmental outcomes [10]. Consequently, several negative repercussions of maternal depression symptoms on child development domains are reported [11]. Specifically, children of depressed mothers report poor cognitive and socioemotional development as well as higher incidents of disruptive behavior. Moreover, maternal depression symptoms during early child development promote children’s maladaptive social cognition resulting in lower social competence in later adulthood [10].

Among mothers with depression symptoms, meta-analysis and observational studies have reported more symptoms, such as negative maternal behaviors, negative maternal affect, and hostile/coercive behaviors as well as disengagement with their infants than among non-depressed mothers [12]. Initiatives for early identification and interventions on maternal depression symptoms during pregnancy and postpartum to improve maternal and child health are underway [9]. However, in low- and middle-income countries, such initiatives are rare despite increasing prevalence of maternal depression symptoms.

Notably, recently a Lancet Series indicated an increasing worldwide burden of mental health disorders including maternal depression symptoms [13]. The Lancet Series’ recommendations clearly indicate the urgency and the role mental health has in most, if not all, of the 17 UN Sustainable Development Goals (SDGs) for 2030, such as SDG 2, 3, and 4, which have an in-depth focus on mental health [13].

In a cluster-randomized controlled trial initiated in 2013, we performed a 6-months’ intervention comprising nutrition, stimulation and hygiene education among impoverished mothers of children aged 6–8 months in rural districts of Uganda [14]. The intervention consisted of educating mothers aimed at (i) increasing complementary foods’ dietary diversity to improve nutrient intake as well as continued breastfeeding, (ii) improving hygiene and sanitation practices, and (iii) enhancing stimulation based on a social-cognitive learning theory to improve development. While this intervention did not alter child growth at the age of 20–24 months, cognitive, language, and motor development improved markedly [14].

We have now examined the possible effects of this intervention on maternal depression symptoms and their associations to child development, among a sub-sample of these mother/child pairs at the child-ages of 12–16, 20–24, and 36 months. In our parental trial, the intervention mothers formed groups that frequently met to practice and share the childcare practices introduced as part of the education intervention. These groups were formed based on the group dynamics theory [15]. According to this theory, groups are known to naturally provide opportunities to develop relationships, help members successfully to accomplish goals, and assist in executing tasks that could not be accomplished individually [15]. Previous studies on small groups indicate that group members enjoy their experiences more when assigned to work in groups rather than by themselves [16].

In this follow-up study, mothers in the intervention group would identify with each other and have shared values, practices and skills on the appropriate complementary feeding taught in the intervention. Moreover, the complementary feeding skills would be learnt through observation, imitation, and positive reinforcement from mother group members. In this follow-up study, we examined if this education intervention (i) would be associated with reduced depression symptoms among the mothers, and (ii) if there were any association between maternal depression symptoms and child development outcomes.

## 2. Materials and Methods

### 2.1. Participants and Approvals

This is a follow-up study of a two-armed, open cluster-randomized education intervention regarding nutrition including focus on complementary feeding, stimulation and hygiene among impoverished mothers of children aged 6–8 months conducted in the Kisoro and Kabale districts of South-Western Uganda [14]. In the current follow-up study, 155 mother/child pairs (77 in the intervention and 78 in the control group) participated. All mothers gave written or thumb-printed, informed consent to participate and could decline an interview or assessment at any time. Exclusion criteria were households with a child having congenital malformation, a physical disorder that would influence assessments and/or nutrient intake, and/or a diagnosis of mental or brain illness, as reported by the mother or a health worker.

The study was approved by The AIDS Support Organisation Research Ethics Committee (no. TASOREC/06/15-UG-REC-009) and by the Uganda National Council for Science and Technology (no. UNCST HS 1809) as well as by the Norwegian Regional Committee for Medical and Health Research Ethics (no. 2013/1833). The parental trial was registered with clinicaltrials.gov (ID no. NCT02098031). We report the data according to the CONSORT guidelines and with intention-to-treat analysis.

### 2.2. Randomisation of the Parental and follow-up Participants

For the parental trial we used proportionate sampling, 10 sub-counties (i.e., clusters) were obtained (6 out of 19 in Kabale and 4 out of 14 in Kisoro). We used a three-stage procedure to identify eligible households. First, by simple random sampling, three sub-counties in Kabale were allocated to the intervention group and the other three to the control group. Similarly, two sub-counties were allocated to the intervention and the other two to the control group in Kisoro. Second, all the villages in each participating sub-county (intervention or control) were listed alphabetically and assigned numbers in an ascending order. By use of computer-generated random numbers, villages to whose assigned number matched with the random numbers were selected. The intervention villages did not share common geographical boundaries with control villages to minimize contamination of the intervention contents between the two study groups. Third, by complete enumeration, all consenting households with children aged 6–8 months within a participating village were recruited to the study. If a household had more than one eligible child, the youngest was selected, and in the case of twins, we randomly selected one for evaluation. We finally enrolled 511 mother-child pairs in the parental study and they were randomized to the intervention (*n* = 263) or the control (*n* = 248) group.

In the current follow up study, the child had to be 12–16 months during the period of January-May 2014 to be included in the this follow-up study. At this stage, group processes of developing relationships among members to successfully accomplish goals would have taken place [14]. Depression data was collected when the children were 12–16, 20–24 months, and at 36 months. Other data was collected when they were 6-8 months (baseline), 12–16, 20–24 months and at 36 months. The data collection teams in the follow-up study were masked to group allocation and never had any interaction with the study team that delivered the education intervention in the parental trial.

### 2.3. The Intervention in the Parental Trial

Details of intervention are reported elsewhere [14,17]. Briefly, the intervention was conducted by the study team at three group meetings over a period of 6 months to 26 groups of mothers (6–10 mothers per group). It was delivered by a trained education team and included two behavior change techniques: Providing information and prompt practice (i.e., demonstrations of preparing food and stimulation of the children). The nutrition education curriculum was based on the 10 guiding principles of complementary feeding [2]. Recipes were formulated and cooking demonstrated using locally available foods with emphasis on protein. Moreover, the need to take ill children to hospital for medical attention and to increase the feeding frequency during and after illness was emphasized. Hand washing before feeding as well as use of clean utensils during food preparation and feeding were parts of the hygiene intervention. A novel aspect of this intervention was the focus on oral hygiene, and distribution of toothbrushes to all household members and demonstration of their use. The education team highlighted the importance of play to improve cognitive, language and motor development. The stimulation intervention was based on social-cognitive learning theory [18]. Based on the group dynamics theory, mothers would share their experiences and practice more of the taught skills as well as assigned to work in groups rather than by themselves [16]. In addition to the three group meetings, the women met at monthly intervals to practice what they had learnt, thus ensuring compliance with the intervention.

### 2.4. Outcomes

The primary outcome of this follow-up study was maternal depression symptoms using Beck Depression Inventory II (BDI-II) scores and Center for Epidemiologic Studies Depression Scale (CES-D) scores. The BDI-II is a self-reported tool for assessing symptoms of depression. It asks mothers to report on a 4-point scale from 0 to 3 with 21 questions, giving a possible range of 0–63 (see: https://www.ismanet.org/doctoryourspirit/pdfs/Beck-Depression-Inventory-BDI.pdf). A score of 10 or above is considered to be indicative of probable depression [19]. Similarly, CES-D asks the mothers to report, on a 4-point scale (0=rarely/none of the time to 3=all of the time), the frequency of symptoms for 20 scale assessment items (see: https://www.outcometracker.org/library/CES-D.pdf). A total score of 16 or higher is considered to indicate depression in the general population [20]. The BDI-II and CES-D have been validated for use in Uganda [21,22]. In addition, CES-D has been used extensively in psychological and epidemiologic studies of postnatal women [23]. Both tools were used because BDI-II has evaluated many different populations in Uganda [19,21], while CES-D has been extensively used in epidemiological studies [22,24]. In the present study, inter-rater reliability for the BDI-II was at least 0.80 across all measurement time points while that for CES-D was at least 0.85. We report scores when the children were 12–16, 20–24 months, and at 36 months. When the parental trial was designed we did not include the BDI-II and the CES-D tools in order not to burden the mothers too much since the education protocol was quite comprehensive and the number of various assessments was large. Consequently, at baseline, we only identified maternal depression symptoms by the following categorized interview question included in our social demographic questionnaire: “How sad did you get with the birth of this child?” Their responses were categorized as not sad (score = 0) or sad (score = 1). After completion of the parental trial, we experienced that the mothers were willing also to undertake a more thorough assessment of their mental health, hence we included the BDI-II and CES-D tools from the 12–16 months’ time point and onwards.

Secondary outcomes included an assessment of whether the maternal depression symptoms were associated with child development, as assessed by the Bayley Scales of Infant and Toddler Development-III (BSID-III) score, which is the most comprehensive development measure for children up to 3.5 years and has been adapted and used in Uganda [24]. Inter-observation agreement was good, as indicated by an intra-class correlation coefficient of 0.75.

### 2.5. Statistical Analysis

In a similar low-resource-setting, Van der Heijden et al. reported a mean difference of about 1.5 SD in BDI-II scores between intervention and control groups [25]. Thus, to calculate the sample size we assumed a difference in BDI-II score of 1.5 SD when the children were 36 months, a power of 0.8 and α of 0.05, hence a minimum of 44 mother/child pairs per group were required. To account for an intra-cluster correlation (ICC) of 0.01 and dropouts, a total of 155 children were included [26]. Among these 155, we randomly selected 77 mother/child pairs from the parental trial intervention group and 78 children from the parental trial control group.

Maternal depression symptoms and child development outcomes were analyzed using Stata/SE (StataCorp. 2015, Stata Statistical Software: Release 14. College Station, Stockholm, Sweden) and SPSS version 22.0 (IBM SPSS Statistics, IBM Corp., Armonk, NY). Significance level was set at *p* < 0.05. According to the skewness test for normality (in the software programme), our data residuals showed a normal distribution. In addition to this, we generated histogram plots, which showed a bell-shaped curve pointing to a normal distribution. We used a mixed effect linear regression to compare the intervention with the control group and estimated ICC. The sub-county (cluster), village, mother, and mothers within villages were the random intercepts, while the time points and group affiliation (intervention or control) were the random slope and fixed variables in the model. Exchangeable variance-covariance structure was used for the random part at the mother level and the models were fitted via the restricted maximum likelihood method. We fitted data by the maximum likelihood method and used a log likelihood-ratio test to determine the overall effect of the intervention for the entire study period. Differences between the two study groups are given as mean (SD or 95% CI).

For the secondary outcomes (association analyses) we used pooled data from the two study groups (intervention and control) to examine associations between maternal depression symptoms using the BDI-II and CES-D scores and the development outcomes by the BSID-III scores using mixed effects linear regression models to handle three data points per mother. In the association analyses, time, maternal depression symptoms’ scores, and interaction were treated as the fixed variables, the sub-county as the cluster and reml as a fitting method. Additionally, these analyses were adjusted for group affiliation.

## 3. Results

### 3.1. Characteristics of the Participants

**Figure 1:**
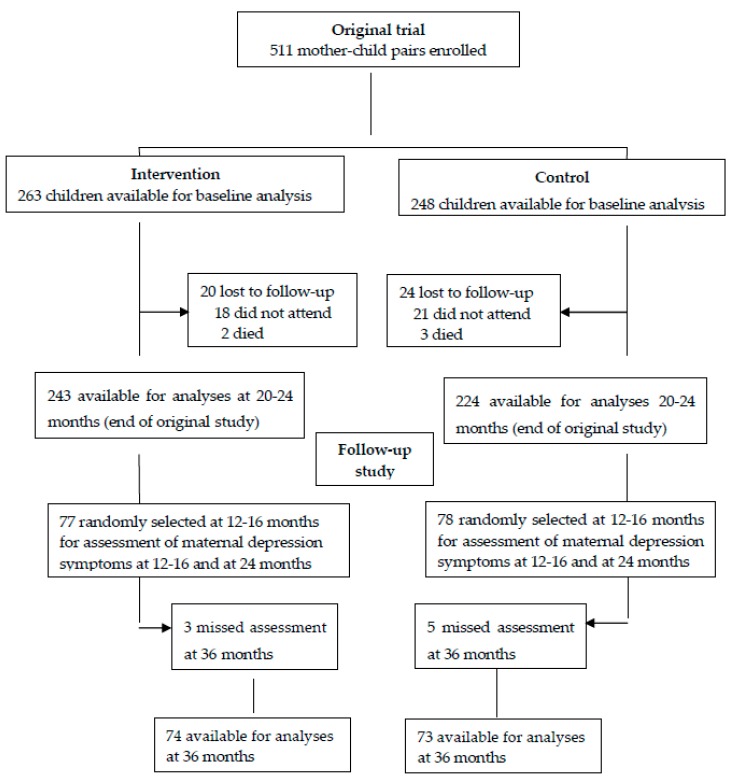
Flow chart of the inclusion process.

The flow chart in Figure 1 shows the inclusion process of the participants in both the parental trial and in the current follow-up study. Of the 511 participants involved in the parental trial, 155 participants were included in the current follow-up study at 12–16 and 20–24 months. By 36 months, eight of them were lost to follow-up (three in the intervention group and five in the control group).

There were no significant differences in the characteristics between the parental cohort (data obtained at baseline) and the follow-up cohort (data obtained at 12–16 months; Table 1), thus no adjustments for baseline differences were made in subsequent analyses.

On the question about maternal sadness asked at baseline, the number (proportion) of mothers in the control group who responded as being sad (score 1) was 43 (55%), while the corresponding value for the intervention group was 40 (52%) (*p* > 0.05).

### 3.2. Effect of the Intervention on Maternal Depression Symptoms

**Figure 2:**
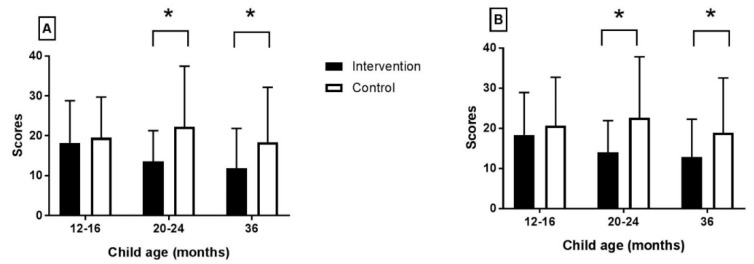
Maternal depression scores derived from the (**A**) BDI-II and (**B**) CES-D questionnaires. Values are means and SD. Asterisk denotes significant difference.

The intervention group mothers had a marked reduction of the depression symptoms at the 20–24 and at the 36 months’ assessment, based on both the BDI-II and CES-D scores (Figure 2, Table 2). In the control group, the depression symptoms had increased at the 20–24 months’ assessment, whereas there was a reduction of the reported symptoms at 36 months. However, the maternal depression symptom scores among the controls were still higher compared to the intervention group mothers.

### 3.3. Associations between Maternal Depression Symptoms and Child Development Outcomes

In the parental trial, we found that the intervention led to better developmental outcomes [14]. We here show that the intervention group had fewer depression symptoms at 20–24 and 36 months based on both the BDI-II and CES-D scores. Therefore, we next examined whether the maternal depression symptoms were associated with the BSID-III child development outcomes. To this end, we pooled the intervention and control groups into one single follow-up cohort (i.e., *n* = 155 mother/children pairs). Notably, the reported depression symptoms assessed by the BDI-II scores were significantly associated with all BSID-III child development outcomes at both 20–24 and 36 months, except the motor child development outcome at 20–24 months (*p* = 0.34; Table 3). At the 36 months’ assessment, all BSID-III child development outcomes were significantly associated with the maternal depression symptoms.

Table 4 shows the corresponding data for associations between the maternal depression symptoms assessed as the CES-D scores. Similar to the results obtained with the BDI-II scores, the CES-D scores were significantly associated with all BSID-III child development outcomes at 20–24 months, except the motor child development outcome (*p* = 0.59). At the 36 months assessment, all BSID-III child development outcomes were significantly associated with the maternal depression symptoms.

## 4. Discussion

In their recent Cochrane report, Arikbo and colleagues found evidence for effects of education interventions on complementary feeding, but no effects on child growth were found and neither developmental outcomes nor maternal depression were evaluated [27]. Moreover, their report only included a few cluster-randomized trials, and none from sub-Saharan Africa. Thus, our study is probably among the first randomized group education intervention trials with a long-term follow-up focusing on nutrition with focus on complementary feeding, stimulation, and hygiene among impoverished mothers of children aged 6–8 months in this African region.

In the parental trial, the 6-months education intervention led to significant improvements in development outcomes when the children reached 20–24 months, but without affecting growth [14]. The follow-up of a sub-sample of the parental trial at 36 months showed a sustained improvement in the development outcomes using three independent tools. Interestingly, the intervention reduced linear growth faltering at 36 months, but had no effect on gut microbiota composition [17].

In the current follow-up study, we found that the education intervention significantly reduced maternal depressive symptoms among intervention mothers compared with the controls. These findings from our randomized trial are consistent with those of interventions focusing on maternal childcare practices. For example, similar findings were reported by Singla et al., whose manualized, parenting intervention in rural Uganda resulted in mothers in the intervention group reporting significantly fewer depressive symptoms than mothers in the control group [24]. Furthermore, a study in Jamaica examining early childhood stimulation intervention among mothers of undernourished children, reported similar findings of reduced maternal depressive symptoms in the intervention group [28].

How our maternal education intervention led to a decrease in the maternal depression symptoms is unknown. In the parental trial we found that the child diet diversity score improved among children in the intervention group [14], and at 36 months, these children had less growth faltering than the control children [17]. Improved nutritional status and/or growth may possibly then have alleviated a burden to mothers in the intervention group who were depressed at study start, so that they later reported fewer depression symptom scores. It has been demonstrated previously that the association between maternal depression symptoms and infant developmental outcomes can inform several interventions to improve early childhood health [29]. Moreover, we cannot exclude that the whole process of forming mother groups based on the group dynamics theory [15] and the subsequent empowering of the intervention women in various ways, may have contributed to a beneficial effect on their depression scores.

Our study findings indicated an inverse relationship between maternal depressive symptoms and child cognitive, language and motor development outcomes. These findings agree with those of observational studies that identified persistent declines in the rate of child development upon exposure to late onset of maternal depressive symptoms [30,31]. A meta-analysis from 2017 based on 14 studies confirmed the association between maternal depressive symptoms and lower cognitive scores among children less than 5 years of age [32]. Moreover, a recent review investigating the various mechanisms through which maternal depressive symptoms are associated with early childhood cognitive development, concluded that the association is linked to family processes and parenting practices [33]. In yet another study, maternal depression symptoms in early childhood were associated with impaired verbal skills in later to middle childhood [34]. Similarly, a Canadian longitudinal study found a direct association between maternal depressive symptoms and child receptive language at ages 4 and 5 years [35].

There is vast evidence on the negative effect of maternal depression symptoms on children’s cognitive, behavioral, and socio-emotional development [33,36]. Our findings of no association between maternal depression and child development outcomes at 12–16 months are also consistent with previous studies. Still, mixed findings are reported in relation to maternal depression effect on child development outcomes among various child age groups. For example, a recent study from the UK concluded that assessing maternal depression symptoms before a child-aged <2 years, is less likely to identify any association than at later ages [37]. Instead, it is the persistent exposure to maternal depression symptoms at 2 years and beyond that will negatively influence child development outcomes during early childhood. In contrast to this, a 2017 meta-analysis of 14 studies showed a statistically significant relationship between maternal depressive symptoms and child cognitive development among children aged 5 years and below [32].

Our results also showed an inverse relationship between maternal depression symptoms and motor development. In support of these findings, previous research reported that maternal depression during early childhood increased the risk of delayed motor (fine and motor) development at three years [38,39].

Collectively, previous research and our current study highlight the importance of early intervention to address maternal depression in early childhood. Specifically, interventions should possibly be directed at educating the mothers regarding the importance of nutrition, hygiene and sanitation as these measures are likely to improve the health of their children and thereby reduce the many burdens mothers in low-resource settings face every day.

This study has several strengths. For example, we adopted a multidisciplinary approach combining aspects of child care practices of nutrition, child stimulation, hygiene, and two independent and validated psychological research instruments for assessing maternal depression symptoms (BDI-II and CES-D) and one well established tool for assessing child development outcomes (BSID-III). Notably, the children were studied for several years with little loss to follow-up and in a randomized controlled trial based in a rural community setting. The study also has some limitations. The use of self-reported maternal depression symptoms could have compromised reporting of as well as mistakes in presenting the symptoms. Notably, the BDI-II and the CES-D tools only provide scoring of maternal depressive symptoms and not a diagnosis of depression per se, although higher scores are considered to be indicating of a depressive disorder as categorized in the ICD-10 and DSM-IV classifications [40,41]. Additionally, we did not assess maternal depression symptoms using the questionnaires (BDI-II and CES-D) at baseline when children were 6–8 months. Lastly, adherence to complimentary feeding as well as possible individual benefits from the formed groups was not assessed in our study.

## 5. Conclusions

Our randomized group education trial focusing on complementary feeding, hygiene, and stimulation education among mothers of 6–8 months old children significantly reduced maternal depression symptoms at child-ages of 20–24 and 36 months. We also found inverse associations between maternal depression symptoms and child cognitive and language development outcomes. The reported positive effects from this intervention would call for further studies of similar interventions in other low-income rural settings before consideration of a larger scale-up in the sub-Saharan region and elsewhere.

## Figures and Tables

**Table 1:** Study population characteristics for the parental trial at baseline and at start of the follow-up study.

Characteristics	Parental Trial (Data Obtained at Baseline)		Follow-up Study (Data Obtained at 12–16 Months)	
	Intervention (*n* = 263)	Control (*n* = 248)	Intervention (*n* = 77)	Control (*n* = 78)
**Children (*n*, %)**				
Males	139 (52.9)	123 (49.6)	44 (57.1)	41 (52.6)
Females	124 (47.1)	125 (50.4)	33 (42.9)	37 (47.4)
Age at inclusion (months)	7.4 (0.8)	7.3 (0.9)	21.4 (1.0)	21.2 (1.0)
**Child growth (*n*, %)**				
Stunting *	55 (20.9)	70 (28.0)	32 (41.6)	46 (59.0)
Underweight *	25 (9.5)	36 (14.5)	6 (7.8)	8 (10.3)
Wasting *	12 (4.6)	12 (4.8)	3 (3.9)	2 (2.6)
**BSID-III composite score**				
Cognitive	114.9 (21.3)	99.3 (17.1)	116.1 (15.6)	105.9 (15.9)
Language	98.3 (14.3)	88.4 (9.1)	106.5 (14.8)	98.9 (12.8)
Motor	113.6 (18.9)	99.1 (14.3)	122.3 (18.7)	113.3 (19.9)
**Maternal data**				
Maternal education (years)	4.9 (2.8)	4.9 (2.8)	5.5 (2.5)	5.0 (2.6)
Maternal age (years)	26.1 (5.8)	26.8 (6.3)	26.2 (6.1)	27.4 (6.4)
Number of children per mother	3.4 (2.2)	3.3 (2.2)	3.4 (2.2)	3.3 (2.2)
**Household data**				
Household head age (years)	31.3 (7.7)	32.6 (19.4)	30.2 (7.3)	33.1 (10.9)
Household head education (years)	6.4 (3.1)	5.9 (3.1)	6.6 (3.3)	6.5 (3.4)
Household size (n)	5.5 (2.1)	5.5 (2.1)	5.7 (2.2)	5.8 (2.2)
Household poverty score	47.8 (11.7)	47.6 (11.4)	49.0 (11.6)	46.3 (12.3)
Sanitation composite score	7.2 (1.9)	7.3 (1.9)	7.0 (1.8)	7.1 (1.9)

Values are means (SD) unless otherwise stated. * based on *z*-score values below 2SD of the median of the reference population. There were no significant differences between the intervention and control groups in either the parental trial or in the follow-up study.

**Table 2:** Mean maternal depression scores derived from the BDI-II and CES-D scales.

	Intervention *	Control *	Inter-Group Difference *	*p*-Value	Overall *p*-Value
	(*n* = 73–77)	(*n* = 74–78)	(*n* = 147–155)		
**Age of Child (months)**	**Beck Depression Inventory (BDI-II)**				
12–16	18.23 (10.55)	19.50 (10.22)	−1.27 (−2.50 to −1.00)	0.48	0.0001
20–24	13.58 (7.70)	22.24 (15.20)	−8.26 (−11.49 to −1.13)	0.0001	
36	11.87 (9.99)	18.41 (13.75)	−6.54 (−8.69 to −2.99)	0.004	
**Age of Child (months)**	**Center for Epidemiological Studies-Depression (CES-D)**				
12–16	18.36 (10.56)	20.67 (12.06)	−2.31 (−4.99 to −1.31)	0.32	0.002
20–24	14.06 (7.85)	22.62 (15.18)	−8.56 (−10.82 to −2.72)	0.0001	
36	12.81 (9.47)	18.90 (13.66)	−6.09 (−9.21 to −3.09)	0.002	

Values are means (SD or 95% CI) of scores and analyzed using linear mixed effect model. * The variation in n was due to missing data because some mothers did not complete all the tests. *p*-value is for the difference between the two study groups at each time point. Overall *p*-value is for the overall effect of intervention obtained from the log likelihood ratio test.

**Table 3:** Associations between maternal depression (BDI-II scores) and BSID-III child developmental scores for the whole study cohort.

Outcome	Child Age (months)	R *	95% CI	*p*-Value **	*p*-Value Interaction ***
**BSID-III scores**					
Cognitive development	12–16	−0.25	−0.30 to 0.20	0.70	0.005
	20–24	−0.30	−0.54 to −0.06	0.01	
	36	−0.31	−0.57 to −0.06	0.017	
Language development	12–16	−0.06	−0.32 to 0.20	0.63	0.031
	20–24	−0.01	−0.15 to 0.13	0.008	
	36	−0.20	−0.23 to 0.16	0.001	
Motor development	12–16	−0.02	−0.25 to 0.02	0.82	0.031
	20–24	−0.11	−0.33 to −0.11	0.34	
	36	−0.29	−0.57 to −0.003	0.043	

* Values are regression coefficients (R) adjusted for group affiliation. At 20–24 months we assessed *n* = 155 children whereas at 36 months *n* = 148 children were assessed. This variation in n was due to incomplete data. ** Mixed effects linear regression *p*-values for the association between maternal depression symptoms and BSID-III child development outcomes. *** *p*-value is the interaction difference between the three time points’ regression coefficients.

**Table 4:** Associations between maternal depression (CES-D scores) and BSID-III child developmental scores for the whole study cohort.

Outcome	Child Age (months)	R *	95% CI	*p*-Value **	*p*-Value Interaction ***
**BSID-III scores**					
Cognitive development	12–16	−0.04	−0.29 to 0.21	0.73	0.026
	20–24	−0.30	−0.04 to −0.001	0.03	
	36	−0.28	−0.53 to −0.04	0.023	
Language development	12–16	−0.05	−0.32 to 0.30	0.52	0.032
	20–24	−0.01	−0.15 to 0.14	0.006	
	36	−0.20	−0.23 to 0.16	0.001	
Motor development	12–16	−0.04	−0.18 to 0.26	0.71	0.025
	20–24	−0.06	−0.27 to 0.15	0.59	
	36	−0.29	−0.55 to 0.03	0.028	

* Values are regression coefficients (R) adjusted for group affiliation. At 20–24 months we assessed *n* = 155 children whereas at 36 months *n* = 148 children were assessed. This variation in n was due to incomplete data. ** Mixed effects linear regression *p*-values for the association between maternal depression symptoms and BSID-III child development outcomes. *** *p*-value is the interaction difference between the three time points’ regression coefficients.
